# Molecular Background, Clinical Features and Management of Pediatric Mastocytosis: Status 2021

**DOI:** 10.3390/ijms22052586

**Published:** 2021-03-04

**Authors:** Magdalena Lange, Karin Hartmann, Melody C. Carter, Frank Siebenhaar, Ivan Alvarez-Twose, Inés Torrado, Knut Brockow, Joanna Renke, Ninela Irga-Jaworska, Katarzyna Plata-Nazar, Hanna Ługowska-Umer, Justyna Czarny, Anna Belloni Fortina, Francesca Caroppo, Roman J. Nowicki, Bogusław Nedoszytko, Marek Niedoszytko, Peter Valent

**Affiliations:** 1 Department of Dermatology, Venereology and Allergology, Medical University of Gdańsk, 80-211 Gdańsk, Poland; hanna.lugowska-umer@gumed.edu.pl (H.Ł.-U.); czarnyjustyna@gmail.com (J.C.); rnowicki@gumed.edu.pl (R.J.N.); bned@gumed.edu.pl (B.N.); 2 Division of Allergy, Department of Dermatology, University Hospital Basel and University of Basel, 4031 Basel, Switzerland; karin.hartmann@usb.ch; 3 Department of Biomedicine, University Hospital Basel and University of Basel, 4031 Basel, Switzerland; 4 Mast Cell Biology Section, Laboratory of Allergic Diseases, National Institute of Allergy and Infectious Diseases, National Institutes of Health, Bethesda, MD 20892, USA; mcarter@niaid.nih.gov; 5 Dermatological Allergology, Department of Dermatology and Allergy, Charité—Universitätsmedizin Berlin, Corporate Member of Freie Universität Berlin, Humboldt-Universität zu Berlin, Berlin Institute of Health, 10117 Berlin, Germany; frank.siebenhaar@charite.de; 6 Instituto de Estudios de Mastocitosis de Castilla-La Mancha (CLMast)—Spanish Reference Center for Mastocytosis, Hospital Virgen del Valle—Complejo Hospitalario de Toledo, 45071 Toledo, Spain; ivana@sescam.jccm.es (I.A.-T.); inestorradoe@gmail.com (I.T.); 7 Department of Dermatology and Allergy Biederstein, School of Medicine, Technical University of Munich, 80802 Munich, Germany; knut.brockow@tum.de; 8 Department of Pediatrics, Hematology and Oncology, Medical University of Gdańsk, 80-211 Gdańsk, Poland; jrenke@gumed.edu.pl (J.R.); nirga@gumed.edu.pl (N.I.-J.); 9 Department of Pediatrics, Pediatric Gastroenterology, Allergology and Nutrition, Medical University of Gdańsk, 80-211 Gdańsk, Poland; katarzyna.plata-nazar@gumed.edu.pl; 10 Pediatric Dermatology Unit, Department of Medicine DIMED, University of Padua, 35128 Padua, Italy; anna.bellonifortina@gmail.com (A.B.F.); francesca.caroppo@outlook.it (F.C.); 11 Department of Allergology, Medical University of Gdańsk, 80-211 Gdańsk, Poland; mnied@gumed.edu.pl; 12 Division of Hematology and Hemostaseology, Department of Internal Medicine I, Medical University of Vienna, 1090 Vienna, Austria; peter.valent@meduniwien.ac.at; 13 Ludwig Boltzmann Institute for Hematology and Hemostaseology, Medical University of Vienna, 1090 Vienna, Austria

**Keywords:** pediatric mastocytosis, *KIT* mutation, tryptase, diagnosis, treatment

## Abstract

Pediatric mastocytosis is a heterogeneous disease characterized by accumulation of mast cells in the skin and less frequently in other organs. Somatic or germline mutations in the *KIT* proto-oncogene are detected in most patients. Cutaneous mastocytosis is the most common form of the disease in children. In the majority of cases, skin lesions regress spontaneously around puberty. However, in few patients, mastocytosis is not a self-limiting disease, but persists into adulthood and can show signs of systemic involvement, especially when skin lesions are small-sized and monomorphic. Children with mastocytosis often suffer from mast cell mediator-related symptoms. Severe hypersensitivity reactions can also occur, mostly in patients with extensive skin lesions and blistering. In a substantial number of these cases, the triggering factor of anaphylaxis remains unidentified. Management of pediatric mastocytosis is mainly based on strict avoidance of triggers, treatment with H1 and H2 histamine receptor blockers, and equipment of patients and their families with epinephrine auto-injectors for use in severe anaphylactic reactions. Advanced systemic mastocytosis occurs occasionally. All children with mastocytosis require follow-up examinations. A bone marrow investigation is performed when advanced systemic mastocytosis is suspected and has an impact on therapy or when cutaneous disease persists into adulthood.

## 1. Introduction

Mastocytosis is a hematopoietic neoplasm characterized by an increase in clonal, morphologically and phenotypically abnormal mast cells (MCs) that accumulate in one or more organ systems [1,2,3,4]. Most commonly, MCs infiltrate the skin, the bone marrow (BM), spleen, liver, lymph nodes, and gastrointestinal tract [1,2,3,4,5,6]. The World Health Organization (WHO) classification of mastocytosis (2016) includes the following categories: cutaneous mastocytosis (CM), systemic mastocytosis (SM), and MC sarcoma (MCS) [3,6]. In CM, MC accumulation is largely confined to the skin, whereas SM is characterized by involvement of extra-cutaneous organs, including the BM [1,2,3,4,5]. SM encompasses indolent SM (ISM), smoldering SM (SSM) and various forms of advanced SM, such as SM with associated hematologic neoplasm (SM-AHN), aggressive SM (ASM) and MC leukemia (MCL). In advanced SM MC infiltrates lead to organ damage [1,2,3,4]. Patients with CM and SM may suffer from MC mediator-related symptoms, including itching, flushing, episodes of hypotension, anaphylaxis, abdominal pain, and diarrhea, among others [5,7,8].

Pediatric patients with mastocytosis usually have CM and only rarely SM [5,7,8,9,10,11,12,13]. The main forms of CM are maculopapular CM (MPCM, termed also as urticaria pigmentosa, UP), diffuse CM (DCM) and mastocytoma of the skin [5]. If SM is present in children, it is mostly diagnosed as ISM [8,9,10,11]. Therefore, mastocytosis in children typically presents as benign disease [5,8,12]. There are no detailed data on the prevalence of childhood-onset mastocytosis. It is estimated to be similar or even higher than in adult-onset mastocytosis, which was found to be 10–13 in 100.000 in the few population-based studies published so far [14,15]. In childhood-onset mastocytosis, a male-to-female sex ratio of 1.4 was reported in a systematic review of 1747 cases [8]. In the vast majority of affected children, disease onset is observed before the age of 2 years, typically in the first 6 months of life [5,8,12,16]. Few patients may already present at birth with mastocytosis [8,12]. Familial mastocytosis and mastocytosis in monozygotic twins have also been reported [5,8,17,18].

In the present study, we have reviewed relevant aspects of the pathogenesis, clinical manifestations and therapy of pediatric mastocytosis.

## 2. Pathogenesis

### 2.1. Genetic Background and Molecular Markers in Mastocytosis

Despite clinical and phenotypic differences between pediatric and adult forms of mastocytosis, both are clonal disorders associated with somatic mutations in a considerable number of cases [16,19,20,21,22,23,24,25,26,27,28]. Somatic gain-of-function mutations in the gene *KIT* result in stem cell factor-independent activation and phosphorylation of KIT, which leads to differentiation of MCs from their hematopoietic stem cells, enhanced MC survival and subsequent accumulation of MCs in various organs [1,2,3,22,23,24]. Therefore, activating mutations of *KIT* are considered to play a crucial role in the pathogenesis of mastocytosis.

In contrast to patients with adult-onset mastocytosis, where the *KIT* mutation D816V in exon 17 is found in over 80% of patients, in children, this mutation is detected only in approximately one third of the patients [8,19,25]. A multicenter French study revealed that 86% of 50 children with mastocytosis had different mutations in *KIT* detected in lesional skin [19]. Mutations of codon 816 in exon 17 (D816V, D816Y and D816I) were found in 42% of the children in this study [19]. The *KIT* D816V mutation was detected in 36% of the cases [19]. Absence of any mutation at codon 816 was found in 58% of the examined children. Sequencing of the entire *KIT* gene in children lacking D816V *KIT* revealed that in 44% of these patients, MCs exhibit mutations outside of exon 17, mainly in exons 8 and 9 [19]. Corresponding results were reported in two recent studies on 11 Japanese and 7 American children with CM [25,26]. Notably, neither clear phenotype-genotype correlation nor relationship between the mutations and familial vs. sporadic disease was found in two French studies on *KIT* mutations in the skin of children with mastocytosis [16,19]. Interestingly, some patients with mastocytosis have no molecular alterations within *KIT,* which suggests that they have alterations in genes other than *KIT* that affect the clinical presentation of the disease [19,22,27].

With few exceptions, mastocytosis is not a hereditary disease. Heritability associated with germline mutations of *KIT* is extremely rare in mastocytosis, and these germline *KIT* mutations occur in different gene regions such as N822I, F522C, K509I, S451C, R634W, Del 419, A533D, S476I, among others [17,29,30,31,32,33,34,35,36,37,38,39,40]. Of note, rare cases of mastocytosis with germline *KIT* mutations associated with tuberous sclerosis and gastrointestinal stromal tumors were reported [35,36,39]. In families in which mastocytosis is inherited in an autosomal dominant pattern, patients usually have increased tryptase levels, extracutaneous involvement and a chronic course of the disease [31,36,37,39].

The mechanisms underlying the correlation between *KIT* mutation and clinical phenotypes of pediatric mastocytosis are poorly understood [16,19,25,26,38,40]. In some sporadic cases with DCM, various somatic *KIT* mutations (D816V, D816Y, D816I, Del419, K509I, internal tandem duplication A502_Y503dup) were reported [19,26,40,41,42,43]. Moreover, in familial DCM, germline mutations such as S451C and A533D were detected [31,37]. *KIT* mutations have also been determined in a few cases of mastocytoma [44]. The sequencing of exons 8, 9, 11, 13, and 17 in skin biopsies from nine cases of mastocytoma revealed *KIT* mutations in six cases (three patients with *KIT* D816V and three patients with internal tandem duplication A502_Y503dup in *KIT* exon 9) [44]. Interestingly, it has been shown that the presence of *KIT* mutations in the skin is not a predictor of disease evolution [16].

The detection of the *KIT* D816V mutation in BM and/or peripheral blood (PB) may suggest the presence of SM [10,11]. So far, however, BM or PB of children with SM were only rarely investigated for *KIT* mutations [45,46,47,48,49,50,51,52,53,54,55]. In addition, *KIT* D816V per se is only a minor SM criterion and detection of the mutation in BM or PB does not automatically lead to the diagnosis SM, neither in adults nor in children. The review of the literature data shows that in two children with SM-AHN, *KIT* D816A and *KIT* D816H were detected [56,57]. Furthermore, in two infants with severe skin involvement corresponding to DCM and one child with extensive skin lesions resembling MPCM, *KIT* D816V was detected in BM samples; one of these patients was finally diagnosed with ASM and the remaining two patients had ISM [7,58,59]. Genetic alterations in children with advanced SM reported in the last decade in the form of case studies are presented in Table 1.

*KIT* mutational status has been determined only in a few cases of MCL and MCS in adults with a previous history of pediatric mastocytosis [29,60,61,62,63,64,65]. The reported patients with MCL showed F522C and Dup502-503 *KIT* mutations, whereas wild-type (WT), Del419, and L799F were detected in those with MCS [61,62,63,64,65]. MCS is a very rare disease. Only a few documented cases of MCS have been reported in children, and in these cases, molecular studies did not reveal *KIT* mutations [66,67,68].

Up to now, more data are definitively needed to further define the clinical relevance of specific genetic alterations reported in childhood-onset mastocytosis. Molecular characterization of the disease will also be of great importance in the future to direct targeted therapy [29,33,43,59,61].

### 2.2. The Impact of Mediators Released by Mast Cells

Clinical heterogeneity and course of pediatric and adult-onset mastocytosis are associated with the effects of certain clinically relevant mediators produced by MCs. These mediators may be released as a result of both IgE-dependent and IgE-independent MC activation [69]. Mediators stored in secretory granules of MCs include, among others, histamine, heparin, tryptase, carboxypeptidase, chymase, and lysosomal enzymes [69]. MCs are also a source of numerous cytokines, chemokines and growth factors. Furthermore, newly formed mediators produced during MC activation include lipids, such as prostaglandin D2 (PGD2), leukotriene C4 (LTC4), platelet-activating factor (PAF), and sphingosine-1-phosphate [69,70,71,72,73,74,75,76,77,78,79,80,81]. The precise role of all these MC mediators in the pathogenesis of CM and SM is not yet fully determined [69]. The best-known MC products mediating symptoms in CM and SM are histamine, tryptase and prostaglandins (PGs) [69,70,71,77,78,79,80]. Local edema, redness, and itching of the skin are related to histamine released from MCs [69,71,77,78]. It was shown that in some patients, MC activation symptoms, including, among others, flushing, pruritus and hypotension, may be accompanied exclusively by excessive release of PGD2 [79]. In recent years, it was found that *KIT* D816V-transformed MCs display also substantial amounts of oncostatin M (OSM), IL-8, chemokine ligand 2 (CCL2), and CCL23 [69,74,82]. MC-derived OSM, for example, stimulates the growth of microvascular endothelial cells, osteoblasts, and fibroblasts [74]. It was also found that CCL2 serum levels are significantly increased in mastocytosis patients compared with controls and higher in patients with advanced SM than in ISM. Based on these data, the hypothesis was raised that CCL2 serves as a novel *KIT* D816V-dependent regulator of angiogenesis and tissue remodeling in SM [75]. Interestingly, it was shown that in children with mastocytosis, MCs may migrate to the skin and BM as the result of upregulation of CCL2/chemokine receptor 2 (CCR2) and vascular cell adhesion molecule (VCAM-1) and they may induce the expression/activation of transglutaminase 2 (TG2) [83]. TG2 is a cross-linking enzyme that promotes the expression of inflammatory cytokines, histamine, and LTC4 [83]. However, additional research is needed to explain the complex impact of MC mediators on the heterogeneous presentation of pediatric mastocytosis.

## 3. Cutaneous Mastocytosis

**Figure 1:**
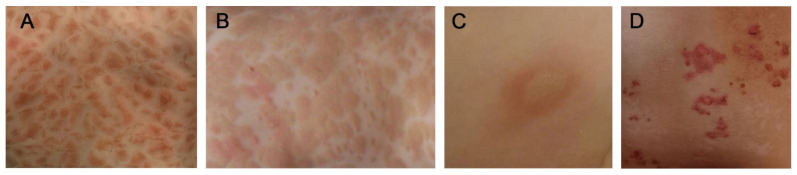
Cutaneous manifestations in Patients with Pediatric Mastocytosis. (**A**) Maculopapular cutaneous mastocytosis—monomorphic variant. (**B**) Maculopapular cutaneous mastocytosis—polymorphic variant. (**C**) Mastocytoma. (**D**) Diffuse cutaneous mastocytosis.

The typical presentation of mastocytosis in children is CM associated with characteristic brown skin lesions [5,84,85,86,87,88]. Specific criteria of CM, defined by the EU/US consensus group, include the absence of signs or criteria of SM, and the presence of typical skin lesions of mastocytosis associated with the Darier’s sign, which is a major CM criterion, and one or two of the following minor criteria: increased numbers of MCs in biopsy sections of lesional skin and an activating *KIT* mutation in lesional skin [5,84]. The Darier’s sign consists of reddening and urticarial swelling of the lesion after mechanical irritation [5]. The generally accepted classification of CM distinguishes three major forms: maculopapular CM (MPCM), diffuse cutaneous mastocytosis (DCM) and mastocytoma of the skin [3,5,6,86] (Figure 1).

### 3.1. Maculopapular Cutaneous Mastocytosis (MPCM)

MPCM is the most common form of CM, regardless of the age at onset of the disease [5,15,87]. In children, the onset is usually within the first 6 months of life [5,16]. Children may present with two variants of MPCM, namely the monomorphic variant and the polymorphic variant [5,88,89,90,91]. Monomorphic MPCM, which is more frequent in adults but can be also observed in a subset of children with mastocytosis, presents with small, round, mostly flat, brown or red maculopapular lesions, which typically show a central, symmetrical distribution on the body and classically spare the central face, palms, and soles [5,88]. Numbers of lesions vary greatly among patients, ranging from several to almost universal coverage [5]. The polymorphic variant of MPCM is, by contrast, almost restricted to children [91]. It is characterized by larger, brown to red heterogeneous lesions of different size, usually with a more asymmetric distribution. Margination, elevation, color, and shape of the lesions can vary among pediatric patients, thus macular, popular, plaque-type or nodular lesions are observed [5,16,88,89,91]. A typical feature of the polymorphic form of MPCM in children, unlike in adults, is the involvement of the head, particularly the lateral parts of the forehead, and the neck and extremities [5,88]. Children with extensive skin lesions may have increased serum tryptase levels in early childhood and more severe MC mediator-related symptoms [5,9,16,90,91]. However, tryptase levels often decrease over time [16,91]. MPCM lesions in children may also evolve during the course of the disease. For example, nodules occurring in infants may change into plaques or papules, often between 5–10 years of age. In general, the skin lesions in pediatric patients often regress around puberty [5,8,9,16,92]. Blistering upon irritation, typical for the infantile period of CM, usually also resolves after 2–3 years of age [5,6,16]. MPCM variants seem to have prognostic impact as polymorphic lesions tend to regularly regress spontaneously around puberty, while in children with the monomorphic variant (adult-type pattern), mastocytosis often persists into adulthood [5,88,92]. In some of these cases with monomorphic skin lesions, SM may be detected in the follow up. It has also been shown that childhood-onset disease with large maculopapular cutaneous lesions, typically representing the polymorphic variant, compared with small lesions, representing the monomorphic variant, is associated with lower serum tryptase levels, a more favorable outcome, with shorter disease duration and more frequent spontaneous remission of skin lesions [92].

### 3.2. Mastocytoma

Mastocytoma is a common clinical manifestation of CM in infants; sometimes it appears at birth, or in young children [5,88,91,93]. Usually, it is a single brown, red or yellow macule or nodule, sharply demarcated from the surrounding area, typically measuring between 1 and 10 cm in diameter [5,93]. This lesion is often located on the trunk but may also be detected in other areas of the skin. In common with all other forms of CM, mastocytomas show the Darier’s sign after rubbing, and may also blister after mechanical irritation or in response to other triggering factors [5,6,93]. Generally, the course of mastocytoma is benign with a tendency to regress spontaneously before puberty. According to the latest recommendations, the term cutaneous mastocytoma may be used in the presence of up to three lesions [5]. When four or more lesions of this nature are found, MPCM is diagnosed.

### 3.3. Diffuse Cutaneous Mastocytosis (DCM)

DCM is a rare and the most severe clinical presentation of CM, associated with pronounced MC infiltration of the entire skin [5,7,88,94,95,96,97,98,99,100,101]. The frequency of DCM ranges from 5% to 13% of all childhood CM forms [7,8,9,12,96]. DCM presents as erythroderma and generalized pachydermia (thickened skin) with pronounced dermographism in the majority of the cases [5]. Extensive spontaneous blistering with erosions is a typical feature of the condition in the infantile period [5,42,80,94,95]. Rubbing or scratching of skin lesions causes the release of MC mediators and results in reddening of the skin, urticarial swelling, and/or blistering. DCM is a clinically heterogeneous disease. The presence of both large hemorrhagic bullous lesions and small vesicles has been reported [94,95]. Less frequently, DCM manifests as generalized erythema with pseudoxanthomatous or large, tumor-like lesions [94,96,98]. Cutaneous manifestation of DCM changes in an age-dependent manner. Extensive bullous lesions predominate in infancy, whereas diffuse infiltration of the skin with hyperpigmentation and leather-like appearance develops in the further course of the disease [5,42,80,94,96,99,100,101]. Therefore, the differential diagnosis of DCM in infants includes numerous bullous skin diseases [80,94]. Blistering usually ceases within 2 to 3 years [5]. Kleewein et al. showed that blistering may be attributable to serine proteases released from MCs and is found within the *lamina lucida* (junctional) [80]. The hemorrhagic character of bullous lesions in children with DCM is probably due to the local release of heparin [5]. DCM is often associated with severe MC mediator-induced symptoms, including flushing, itching, blistering, hypotension, and sometimes even anaphylactic shock [7,12,41,94]. The true incidence of anaphylactic shock in DCM is difficult to estimate because of the rarity of the entity [94,95]. Little is also known about the true frequency of SM in patients who initially present with DCM [7,41,58,94,95,96,97]. It was found that children with skin manifestations of DCM may suffer from WDSM which refers to SM displaying mature MC morphology in the absence of strong CD25 and CD2 expression, and no *KIT* D816V mutation in BM in most cases [97]. There was no case of SM in a series of 14, 10, and 8 children with DCM reported in the literature [9,94,95]. Interestingly, *KIT* D816V mutation in PB was not detected in 10 DCM children checked for SM [10]. Furthermore, in two recently reported cases of DCM, presenting with extensive bullous lesions and serum tryptase level over 100 ng/mL, SM was not diagnosed [42,101]. Nevertheless, rare cases initially diagnosed with DCM turn out to have MC infiltration in the liver, spleen, lymph nodes and BM, leading to chronic diarrhea, malnutrition; in some of these cases an associated myeloproliferative disorder was reported [58,102,103,104]. The majority of infants with DCM have markedly elevated serum tryptase level due to massive MC infiltration of the entire skin [16,94]. In most of these patients, increased serum tryptase level at presentation declines over time and correlates with clinical improvement of cutaneous lesions and MC mediator-induced symptoms [9,16,94].

## 4. Mast Cell Mediator-Related Symptoms

Symptoms related to MC mediator release in children with mastocytosis are highly heterogeneous, ranging from mild itching to severe anaphylaxis [5,7,8,12,87,88,90,105,106,107,108,109,110]. Nevertheless, a significant proportion of patients are fully asymptomatic or present with minimal symptoms. Overall, the type, frequency and severity of MC mediator-induced symptoms depend on the extent of tissue involvement and serum tryptase levels [5,7,90,107,111]. Early recognition of the signs and symptoms following MC activation may be crucial to select appropriate treatment and to avoid further complications, particularly among children with extensive cutaneous disease and/or increased serum tryptase levels [112]. Despite this, symptoms tend to improve over time and finally regress in most patients, regardless of the severity of symptoms and the extent of skin involvement in early childhood [5,16,87].

### 4.1. Cutaneous Symptoms

Skin MC mediator-related symptoms are the most frequent manifestations in children with mastocytosis, regardless of the subtype of CM. These include pruritus, redness and swelling of skin lesions, flushing, blistering and dermographism. Once dermal MCs are activated, skin lesions become erythematous, edematous and pruritic, sometimes also followed by the emergence of vesicles as well as serous or hemorrhagic blisters, particularly within the first 12–24 months from disease onset [5,7,12,87,88]. Tryptase levels in the blister fluid can exceed 80,000 µg/L in highly severe cases, which reflects the extent of the underlying activation of MCs and degranulation process, particularly in children with DCM [7]. In line with this, extensive blistering has been regarded as an indicator of massive MC activation potentially leading to severe complications in pediatric mastocytosis [111]. Other products of MC activation that have also been detected in blister fluid from children with mastocytosis include PAF, PGD2 and histamine [113]. Flushing in CM usually manifests as a sudden reddening of the face and, less frequently, the upper trunk which is caused by an increased blood flow through the skin as a result of vasodilation of dermal capillaries secondary to vasoactive substances released by MCs. Thus, although flushing is widely considered a cutaneous manifestation in nature, its development corresponds to an underlying systemic vascular response to MC mediators which can also lead to a hypotensive collapse in some cases [7]. For this reason, the presence of flushing in a child with mastocytosis should be regarded as a warning sign indicating the need for urgent antimediator therapy and a close watchfulness of the patient. It is worth noting, however, that exercise is a common trigger of flushing and typically not associated with hemodynamic compromise. Overall, episodic flushing with or without other MC mediator-related symptoms occurs in up to 30–50% of children with MPCM, particularly in those with extensive skin involvement, and in virtually all patients with DCM [5,7,12,87,88,90,109].

### 4.2. Symptoms Resulting from Extracutaneous Involvement

In contrast to the high incidence of cutaneous manifestations, symptoms involving organs other than the skin are relatively uncommon in children with mastocytosis. This low frequency of extracutaneous symptoms fits with the fact that mastocytosis in the pediatric population is mostly restricted to the skin [5,8,110,114]. Despite this, some children with mastocytosis may present with a wide range of systemic symptoms, such as abdominal cramping, diarrhea, vomiting, nausea or reflux even in the absence of systemic involvement [8,12,87,90,114]. Thus, these symptoms might be due to the effects in the gastrointestinal tract of high concentrations of MC mediators massively secreted by dermal MCs, rather than increased intestinal MCs [110]. Overall, the frequency of gastrointestinal complaints varies from 15% to more than 50% in children with CM [7,12,87,88,90,107,110]. Other extracutaneous MC-mediator symptoms which are relatively common in adults with mastocytosis such as headache, fatigue, bone pain or cognitive complaints (e.g., “brain fog” or lack of concentration) are rarely seen in children. Only one study examining 28 children with mastocytosis has shown an association with a concomitant diagnosis of autism spectrum disorder (ASD) and reported a higher prevalence (10 times) of autism, Asperger’s disorder and pervasive neurodevelopmental disorder when compared to the general public [115]. This data was obtained by a parental survey of 400 patients and may not reflect the precise prevalence of such entities [115]. Interestingly, cytokines released from MCs such as tumor necrosis factor alpha (TNF-α) or interleukin-6 (IL-6) have been suggested to play a role in the pathogenesis of ASD [115].

### 4.3. Anaphylaxis

Overall, the risk of anaphylaxis is lower in patients with pediatric mastocytosis compared to adult-onset mastocytosis (<1–9% vs. 35–50%) [7,8,106,107,108,109,116]. In contrast to adults in whom the severity of mediator-related symptoms may be associated with SM in the absence of cutaneous lesions (i.e., BM mastocytosis, BMM), anaphylaxis in pediatric mastocytosis typically occurs in patients with extensive cutaneous disease (i.e., >90% of body surface area involved), regardless of the existence or absence of an underlying systemic disease [90,97,107,108,110,117,118,119]. Despite this, anaphylaxis may also occur in children with large solitary mastocytoma, mostly after stroking or rubbing the lesion [5,120]. Anaphylaxis in children with mastocytosis seems to be more prevalent in those with massive infiltration of dermal MCs. Rarely, anaphylaxis may also occur in children with MPCM presenting with a more limited skin involvement and normal serum tryptase levels [109]. Another difference between adults and children is the frequency of triggers of anaphylaxis. Thus, the most frequent elicitor of anaphylaxis in adult patients with mastocytosis is hymenoptera venom, followed by drugs and foods [106,107,116,117,121]. By contrast, the frequency of hymenoptera sting anaphylaxis in pediatric mastocytosis is virtually nil, cases of anaphylaxis being mostly idiopathic in nature [106,107,109]. Among identified causes of anaphylaxis in children, food intake, rubbing of skin lesions, heat, fever, irritability and vaccines are the most relevant triggers [97,106,107,108]. In turn, it should be noted that common medications such as nonsteroidal anti-inflammatory drugs (NSAIDs) and anesthetic procedures represent very rare elicitors of anaphylaxis in pediatric patients [122,123,124,125,126].

## 5. Systemic Mastocytosis

The review of the literature indicates that SM is rarely found in children [7,8,12,19,127]. However, several recent studies based on groups of children with manifestations of CM show that SM is lately diagnosed more frequently than at the beginning of last decade [7,8,9,10,11,12,19] (Table 2). This might be mainly due to the progress in the diagnostic work-up which has been made in recent years, particularly as far as identification and quantification of the *KIT* D816V mutation in PB are concerned [128,129].

The diagnostic criteria of SM in children are the same as in adults. The major criterion of SM is the histological demonstration of multifocal dense infiltrates of MCs (at least 15 MCs/aggregates) in the BM biopsy and/or in sections of other extracutaneous organ(s). Minor SM criteria include: a. >25% of all MCs are atypical cells (type I or type II) on BM smears or are spindle-shaped in MCs infiltrates detected on sections of visceral organs; b. *KIT* point mutation at codon 816 in the BM or other extracutaneous organ(s); c. MCs in BM or blood or other extracutaneous organ(s) exhibit CD25 and/or CD2; d. baseline serum total tryptase level >20 ng/mL (in case of a concomitant myeloid neoplasm AHN, an elevated tryptase does not qualify as SM criterion) [3]. The diagnosis of SM can be established when the major and at least one minor criterion or, in the absence of the major criterion, 3 minor criteria are fulfilled.

The majority of children with SM suffer from ISM with skin involvement, usually in form of the MPCM variant [7,10,11,12,50,52,59,130]. Advanced forms of SM such as ASM, SM-AHN and MCL, are extremely rare in childhood-onset mastocytosis and thus are usually published in the form of case studies [45,48,51,53,54,55,56,57,58] (Table 1). Children with advanced SM may present with skin lesions corresponding to MPCM or DCM [53,58]. However, most reported children with advanced SM fail to show typical skin lesions which may lead to a delay in the diagnosis [45,48,51,54,55,56,57]. One should keep in mind that also adults with advanced SM frequently do not present with cutaneous lesions and symptoms [85]. Furthermore, some children with advanced SM may have no *KIT* D816V mutation in BM [45,51,53,54,55].

## 6. Diagnosis

**Figure 2:**
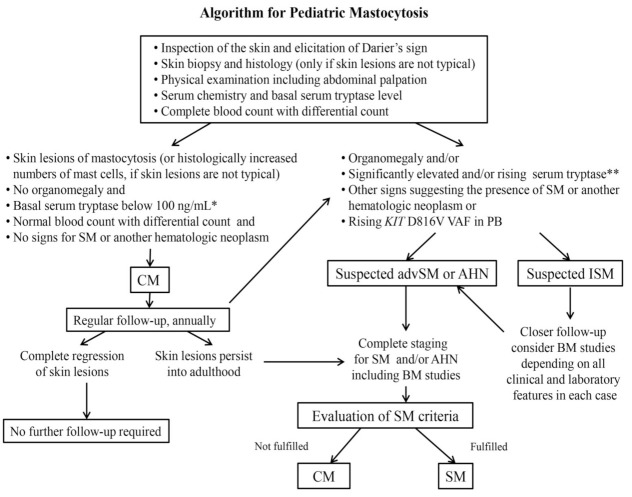
Diagnostic algorithm for Pediatric Mastocytosis. In most pediatric patients with skin lesions, no signs and symptoms indicative of the presence of SM or another systemic hematologic neoplasm is found in initial investigations so that the final diagnosis is CM (left part of the algorithm). In these patients no BM investigations is performed unless the skin lesions persist into adulthood. In a few pediatric patients, however, blood count abnormalities, organomegaly and/or other findings suggest the presence of SM or another hematologic malignancy (right part of the algorithm). Depending on the abnormalities detected, these patients are more closely followed (suspected ISM) or undergo a complete staging including BM studies. Abbreviations: CM—cutaneous mastocytosis; SM—systemic mastocytosis; ISM—indolent SM; advSM—advanced SM; AHN—associated hematologic (non-mast cell) neoplasm; PB—peripheral blood; VAF—variant allele frequency; BM—bone marrow; * elevated serum tryptase level (20–100 ng/mL) should be assessed in correlation with the intensity of skin involvement and in the context of a known hereditary alpha tryptasemia; ** significantly elevated serum tryptase level is not in itself the indication for performing BM studies.

The diagnosis of CM is based on the morphology of skin lesions, the positive Darier’s sign and the histological examination of skin lesions stained for tryptase and/or CD117 [5,114]. In patients with CM, the average numbers of MCs are increased about 3- to 8-fold in the lesional dermis compared with normal skin in healthy subjects (around 40 MCs/mm^2^) and about 2- to 3-fold compared with those suffering from inflammatory cutaneous diseases [5]. It is important to point out that the presence of the *KIT* D816V mutation in lesional skin confirms the diagnosis of CM, but it is not a diagnostic criterion of SM. Taking into account that SM in children is a rare finding, a generally accepted approach is to consider BM investigation only in selected children where advanced SM is suspected. In these children the burden of MCs and thus the risk of severe events is very high (example: rapidly increasing serum tryptase levels) and, therefore, the diagnosis of SM would change the approach to therapy [1,2,3,4,110]. If SM is suspected, *KIT* D816V mutation analysis (*KIT* D816V ASqPCR) in PB has recently also been recommended [10,131]. In the remaining children who fulfill CM criteria and have no other abnormalities or definitive signs of SM, the final diagnosis CM can be established without BM investigation and *KIT* mutation analysis [5,85,110,114,132] (Figure 2).

As mentioned above, the diagnosis of SM in children is based on the same criteria as in adults [1,2,3]. The decision of when to perform BM examination in a child with cutaneous lesions can be difficult. The rare occurrence of SM in children as well as the fact that a BM biopsy is an invasive procedure, with a low, but measurable risk of side effects and emotional stress should be taken into consideration. In recent years, the application of sensitive, allele-specific quantitative polymerase chain reaction (ASqPCR) *KIT* D816V mutation analysis in PB has become a standard screening examination in adults with manifestations of CM and those with suspected mastocytosis without cutaneous signs and symptoms [128,129,132]. As far as pediatric mastocytosis is concerned, Carter et al. (2018) showed that detection of *KIT* D816V in PB of 65 children with all forms of CM and ISM strongly suggests systemic disease [10]. On that basis, *KIT* D816V ASqPCR in PB has been incorporated into a recent diagnostic algorithm of pediatric mastocytosis [10,131]. It has been proposed to use this analysis in children with organomegaly, elevated tryptase and/or severe MC mediator-related symptoms and extensive skin involvement [10,11,131]. The determination of *KIT* D816V mutation in PB is of great importance to identify the subgroup of children who are at risk of SM, need more careful follow-up and further BM investigation in selected cases [10]. However, it should be underlined here that the result of a *KIT* D816V mutation analysis in PB of children should be interpreted cautiously and in combination with other clinical data, particularly organomegaly, serum tryptase levels and PB count [10,11,110]. In addition, as mentioned before, *KIT* D816V per se is only a minor SM criterion (even when detected in PB and BM) but does not automatically lead to the diagnosis SM. Patients with manifestations of SM and negative *KIT* D816V mutation should be evaluated for other mutations in *KIT* [10].

Nowadays, apart from *KIT* D816V ASqPCR in PB, organomegaly is the most reliable clinical predictor of SM in children [7,9,10,110,131]. It was shown that children with elevated serum tryptase level, but no organomegaly, did not fulfil SM criteria [9]. Furthermore, it was reported that increased serum tryptase levels in small children are commonly related to extensive skin involvement (particularly in children with DCM) without underlying SM [7,94,109,133]. In children with CM, serum tryptase levels usually decrease over time and correlate with the improvement of clinical symptoms [9,91,94,109]. On the contrary, persistently very high serum tryptase levels (>100 ng/mL) or a steady increase in basal tryptase level suggest either disease progression, or the development of a non-MC lineage BM neoplasm [85]. Therefore, serum tryptase levels, PB counts and biochemical blood parameters should be determined at first evaluation and at follow-up visits. Basal serum tryptase level is the only SM criterion which can be easily measured in serum outside of highly specialized centers and, for this reason, it is a valuable diagnostic tool. In addition, an acute, event-related increase in serum tryptase levels above the individual’s baseline is a reliable marker of severe systemic MC activation and thus anaphylaxis. In this context, it is worth noting that it is essential to also collect serum samples for basal tryptase level measurements at least 24 h after complete resolution of all anaphylaxis-related symptoms [85]. Of note, elevated serum tryptase is not disease-specific for mastocytosis, because it may also be elevated in patients with chronic urticaria, kidney failure, chronic helminth infections, hereditary alpha-tryptasemia, or other myeloid haematological diseases [1,2,132,134].

In children with highly suspected SM, further diagnostic steps include histological and immunohistochemical examination of BM with staining for tryptase, CD117, CD25, CD2, and CD30, evaluation of BM smear using Wright-Giemsa or May-Grünwald-Giemsa stain, flow cytometry (same markers as above) and determination of *KIT* D816V mutation in BM according to the same standards as previously described in detail for adults [1,2,3,4,85]. All children with cutaneous lesions typical for mastocytosis and significant abnormalities in PB should be referred to hematologists to exclude or establish the diagnosis of SM, assess the BM MC burden, and rule out or diagnose an associated hematological neoplasm (SM-AHN). Moreover, flow cytometric studies, chromosome analysis, additional fluorescence in situ hybridization studies, and next generation sequencing (NGS; myeloid panel) should be performed in cases of suspected SM-AHN [1,2,3,4,132]. In children with SM further stratification needs to be performed to define the type of the disease (ISM, SSM, SM-AHN, ASM, MCL) on the basis of evaluating possible B-findings and C-findings, as described in detail for adult-onset SM [1,2,85,132]. In those children in whom cutaneous lesions persist into adulthood, all SM criteria should be re-evaluated [85].

## 7. Treatment of Pediatric Mastocytosis

### 7.1. Avoidance of Triggers

Parents and caregivers of children with mastocytosis should be informed about environmental factors that can cause symptoms through the release of MC mediators [13,135,136,137,138,139]. Triggers vary greatly from patient to patient, and include mechanical or other physical stimuli, infections, teething, allergens, and drugs [7,12,90,106,107,108,109,110,125,126,135,136,137,138,139,140] (Table 3). Simple measures such as avoiding friction on affected skin area, heat exposure and sudden temperature changes, can have a significant impact on mitigating symptoms and preventing exacerbations [101,136,137]. Various drugs have been considered as potential triggers of MC activation. These include, among others, NSAIDs, opioids, muscle relaxants, quinolones, succinylcholine, agents with tetrahydroisiquinoline, and cough suppressants [123,132,135,137,139,140]. However, significant reactions to these agents are rare. Abstinence from certain drugs, like aspirin and other NSAIDs in particular, is often recommended although data from controlled studies are lacking [140]. Whenever possible, agents with a low risk profile for hypersensitivity reactions should be prescribed and novel drugs should be introduced with caution.

The risk of unforeseeable reactions, for example due to anesthesia, can be reduced through an accompanying premedication (H1-/H2-antihistamines 1–2 h, prednisone 12–24 h/1–2 h prior intervention, and/or sedatives as required) [122,124,125,137]. The most important action to be taken by parents to prevent any major complication in their children during a medical intervention is to inform all involved physicians, including the surgeon and anesthetist, about the disease, previous events (if any) and the related risks.

### 7.2. Emergency Medication

The prescription of an epinephrine autoinjector is generally recommended for adult mastocytosis patients. In children, the decision to prescribe an “Epi-Pen” is based on symptoms, course, and subtype of CM. Current recommendations suggest to provide the Epi-Pen to children with extensive skin lesions, a history of severe systemic symptoms or anaphylaxis, and highly elevated serum tryptase levels [108,131,132,135,136,137,138,139]. In case of severe anaphylaxis, epinephrine is dosed by body weight at 0.01 mg per kg of body weight to a maximum dose of 0.5 mg. When using epinephrine auto-injectors, children weighting between 7.5 and 25 kg should be given the 0.15 mg dose, whereas those with a body weight over 25–30 kg should be equipped with the 0.3 mg dose [141]. Parents, elder children as well as their caregivers in kindergarten, (pre-) school, sports club, etc. should be informed and trained when and how to administer the auto-injector. In some countries, it is also recommended to prescribe a second epinephrine auto-injector for patients with MC disorders, which, if necessary, can be used after at least a 5 min interval from the administration of the first auto-injector [142].

### 7.3. Anti-Mediator Therapy and Mast Cell-Targeted Treatment Options

As of yet, no curative drug treatment options for mastocytosis are available. In most children with CM or SM, no intensive therapy is required. Rather, the major goal in these patients is symptom control, which is based on drugs that inhibit MC activation, prevent release of mediators and block their receptors [135,136,137,138,139]. Treatment modalities depend on the nature, the intensity as well as the frequency of symptoms, and focus on the use of second-generation H1-antihistamines (sgH1-AH) in a step-up approach—Table 4. Despite the lack of controlled studies in children, it is recommended and considered safe to increase the daily dose up to four times the weight-adopted standard dose [132,135,139]. In case of frequently recurring symptoms, a regular given dose split into half twice daily is preferred over an on-demand treatment. If symptoms persist, add-on of H2-antihistamines and/or cromolyn may be considered, especially for gastrointestinal symptoms, and leukotriene antagonists may also be considered in resistant cases [49,131,135,137]. Omalizumab, a monoclonal anti-IgE antibody, was shown effective as a third-line treatment option for mastocytosis, especially in patients with severe recurrent anaphylactic episodes [131,137,143,144,145,146,147]. In-label-use of omalizumab includes chronic spontaneous urticaria (≥12 years) and allergic asthma (≥6 years).

In general, cytoreductive therapy or targeted drugs, such as *KIT*-targeting tyrosine kinase inhibitors (TKI) are not recommended in childhood-onset mastocytosis as the disease is usually indolent with good prognosis [110,131,132,135]. However, use of imatinib associated with improvement of skin lesions has been reported in few children with CM without a *KIT* mutation at codon 816 [43,148]. In one study, reporting on two children with DCM, severe mediator-related symptoms that failed to respond to other treatments and presence of the *KIT* mutation Asp419del in exon 8, treatment with imatinib induced rapid improvement of cutaneous lesions within few weeks [43]. It can be assumed that spontaneous resolution of skin lesions, without administration of imatinib, would have probably taken longer in these two patients. The other study described an infant with MPCM that also showed improvement of cutaneous lesions in response to imatinib, although it can be discussed in this case whether treatment with imatinib was really indicated and whether similar improvement would have also occurred spontaneously [148]. In these few cases, imatinib was rather well tolerated, with occurrence of neurologic hyperexcitability in one patient [43]. It is worth noting that imatinib also exerts profound effects on normal myeloblasts and normal MCs, sometimes resulting in skin depigmentation and MC deficiency. Some of the clinical effects described above may have resulted from such imatinib effects. Another important aspect is that imatinib may lead to growth retardation in children. All in all, imatinib should only be administered with caution in pediatric patients.

The treatment of SM in children lacks standard recommendations and must be based on the type and severity of symptoms, age, the category of SM and the presence of co-morbidities. Midostaurin, a multi-kinase inhibitor, is FDA-approved for the treatment of advanced SM and FLT3-mutated acute myeloid leukemia (AML). Recently, effective and well-tolerated therapy with midostaurin in an infant with ISM has been reported [59]. The child had extensive skin lesions with numerous bullae, highly elevated serum tryptase (267 ng/mL), *KIT* D816V mutation in the BM, anemia, thrombocytosis, and low albumin. In this patient, initial anti-mediator therapy, including antihistamines, cromolyn, montelukast, and methylprednisolone was not sufficient to achieve significant clinical improvement [59]. At age 7 months, treatment with midostaurin was started at increasing doses of 30, 45, and 60 mg/m^2^ twice daily with prophylactic ondansetron given before each dose. After 12 weeks of treatment, the patient entered significant clinical improvement with total resolution of blistering and a decrease in serum tryptase level [59]. Overall, the therapeutic options for the treatment of advanced SM (ASM, SM-AHN and MCL) in children are similar to those in adults [1,2,3,131,132,137,138]. In recent years, treatment with TKI, poly-chemotherapy and allogenic hematopoietic stem cell transplantation, has been reported in occasional cases of advanced SM in children [54,55,57,58].

### 7.4. Vaccination in Children with Mastocytosis

Children with mastocytosis should be vaccinated according to local institutional or national recommendations. Hypersensitivity to vaccines in children with mastocytosis is not considered to be significantly higher than that in the general population (3–6%) and reactions are usually mild and transient [131,149,150]. Nevertheless, it is reasonable and should be recommended to patients to have vaccinations done by an experienced center and to observe the child post-vaccination for 1–2 h [131]. In the event of an adverse vaccine reaction, patients should be referred to an allergist to investigate possible vaccine components (i.a. gelatin or latex) as an inciting agent [151].

### 7.5. Topical Treatment Options and Phototherapy

Topical treatment of CM in most pediatric patients is subordinate to oral antimediator therapy. It fulfils a supporting function in children who suffer from dryness of the skin, pruritus or blistering. In children with dry skin emollients soothe the skin, prevent dehydration of the stratum corneum, and thereby help to reduce the itch and the sensitivity to physical stimuli [110,137]. Disodium cromoglycate at a concentration of 1% to 4% in aqueous solutions or mixed into water-based emollient cream may decrease itching [137,152,153].

Topical steroids are used to avoid MC degranulation in children with extensive skin lesions and recurrent blistering [42,137]. Therapy with topical antibiotics such as mupirocine or fusidic acid can be considered in children with denuded skin erosions in order to avoid skin infections [108]. In infants, mild or medium potency corticosteroids are preferable. The effective therapy with mometasone furoate 0.1% cream in a neonate with DCM whose skin was diffusely red-brown, infiltrated, with many hemorrhagic bullae, crusts, and urticarial lesions was reported [42]. However, topical corticosteroids should be used only over short-term periods for limited areas of the skin due to the risk of skin atrophy and adrenal suppression [42]. High potency corticosteroids (clobetasol propionate 0.05%) under occlusion may be occasionally considered for treatment of solitary mastocytoma [90]. A retrospective study of 133 children revealed that mastocytoma lesions improved after 4 years of observation in 99 patients, equally in the treated and untreated groups, but the time of healing was shorter when topical corticosteroids were applied [154]. In children with mastocytoma in whom mechanical irritation of the lesion provokes flushing or hypotension resistant to antimediator therapy, surgical excision may be considered [93].

Calcineurin inhibitors, particularly pimecrolimus, can be used in children with severe or highly bothersome skin lesions [155,156]. In one study which was, however, conducted without a control group, topical therapy with 1% pimecrolimus cream was applied twice daily on MPCM and mastocytoma lesions in 18 pediatric patients [155]. The mean duration of therapy was 8.3 months for each patient. In some of the children, the lesions faded, and few lesions even disappeared. Clinical evaluation 12 months after cessation of therapy showed no relapse of the lesions that had disappeared [155]. It can be discussed, however, whether the observations in this study rather reflect, at least in part, the natural course of pediatric mastocytosis typically showing spontaneous resolution. Nevertheless, due to its low percutaneous absorption and the lack of side effects associated with corticosteroids, pimecrolimus might be considered as one of the therapeutic options for CM in severely symptomatic children.

Narrow-band ultraviolet B (NB-UVB, 311 nm), ultraviolet A (UVA) and UVA in combination with psoralen (PUVA) have been used for therapy of cutaneous symptoms in adult patients with mastocytosis who suffer from extensive skin lesions [157,158,159]. Both NB-UVB and PUVA induce relief of the pruritus and decrease the visibility of the skin lesions [158]. Nevertheless, phototherapy is not generally recommended in pediatric patients with mastocytosis, because of the tendency towards spontaneous regression of skin lesions around puberty. Moreover, the overall period of symptom reduction is relatively short, and little is known about the long-term safety of phototherapy or psoralen exposure in children [135,160]. The exact mechanism of ultraviolet action in CM is not fully understood [161,162]. Some studies indicate that UVA reduces the numbers of MCs in lesional skin and urine histamine levels [157,163]. UVB may suppress histamine release from MCs and decreases serum tryptase levels [164]. An important aspect of the UV mechanism of action relies on inducing apoptosis in skin cells [165]. PUVA therapy may be considered in selected children with extensive skin lesions and severe MC mediator-related symptoms which do not respond to anti-mediator therapy [136,166,167]. Nevertheless, special caution must be taken when considering PUVA therapy in children because it is associated with the risk of developing skin cancers (squamous skin carcinoma and melanoma) in later life, cataract, and the hepatotoxicity of psoralen. NB-UVB therapy has no significant influence on a higher risk of skin cancers and is free of side effects of psoralen [168]. However, considering the lack of controlled studies and the more vulnerable skin structure in children, the risk of long-term side effects of NB-UVB therapy may be much higher in pediatric than in adult patients.

## 8. Prognosis

In general, the prognosis for pediatric mastocytosis is favorable due to the tendency towards spontaneous regression of skin lesions, which is observed in most children around puberty [8,126,130,169,170]. Overall, anaphylaxis and systemic involvement are rare in childhood CM, although MC mediator-related symptoms are frequently seen in cases with DCM [8,9,10,11,106,107,108,109,110,111,126,130]. Recent studies on the natural course of childhood-onset mastocytosis suggest that complete regression of skin lesions is mostly seen in patients with mastocytoma and MPCM [8,169]. In one study, the complete regression rate for mastocytoma was estimated to be 10% per year, the rate for MPCM/UP was 1.9% per year, whereas there was no evidence of complete regression in cases with DCM and SM [169]. In another study, however, spontaneous decrease of skin lesions was reported in 5 out of 12 patients with childhood-onset DCM [92]. Generally, the prognosis for DCM is uncertain because severe, life-threatening MC mediator-related symptoms may occur in the infantile period. Moreover, low tendency to complete regression of skin lesions is reported, and it is difficult to predict the risk of SM due to the very rare occurrence of DCM [41,42,58,94,95,100,101,102,103,104]. Interestingly, in a group of 15 adults with a previous history of childhood-onset mastocytosis diagnosed 20 years earlier, complete regression was reported in 67%, major regression in 20%, and partial regression in 13% [130]. In most children with ISM, the prognosis is good, whereas in advanced SM and MCS, the prognosis is poor or very poor, similar to adults [53,55,56,57,58,64,66,67].

For many years, studies on prognostic factors for pediatric mastocytosis have been conducted. It was found that the presence of a *KIT* mutation in the skin as well as the immunophenotype of skin MCs (expression of CD25, CD2, CD30) do not correlate with the clinical form and course of CM [16,171,172]. An early-onset of the disease (under 2 years), a polymorphic variant of MPCM, and a small number of skin lesions seem to be associated with a more favorable outcome in the majority of children with CM [16,91,92,126,170]. The prognostic value of *KIT* D816V mutation in PB requires further studies [10,11].

## 9. Concluding Remarks

Children diagnosed with mastocytosis have a good prognosis in comparison to adult-onset disease and rarely progress to a more severe variant. Complete resolution has been documented and moderate and partial resolutions are common in childhood mastocytosis. When compared to adults, children have fewer triggers of MC activation and are mainly managed symptomatically. However, in patients with DCM, mediator-related symptoms and anaphylaxis are frequently seen and represent a clinical challenge. Furthermore, in children with CM, transition to SM is rare and progression to advanced SM is very uncommon. When more aggressive therapy is required to bring MC expansion or MC activation under control, a multidisciplinary approach is recommended. Studies in children are needed to document the occurrence of other diseases in patients with mastocytosis. Moreover, larger multicenter studies on childhood-onset mastocytosis are necessary to develop more specific recommendations for pediatric mastocytosis.

## Figures and Tables

**Table 1:** Advanced SM in children: case studies with molecular diagnostics published in the last decade.

Reference	Diagnosis, Category of SM	*KIT* Mutation in BM	Cytogenetics, Molecular Diagnostics	Age Sex	Cutaneous Involvement, Type of Lesions
Intzes S. et al. (2011) [45]	SM-AHN (AML)	D816V (−)	t(8;21)(q22;q22)	5 years F	no
Mahadeo K.M. et al. (2011) [51]	SM-AHN (AML)	D816V (−) No mutation in exon 8, 9, 11, 13, 17	t(8;21)(q22;q22)	10 years F	no
Yabe M. et al. (2012) [56]	SM-AHN (AML)	D816A (+)	t(8;21)(q22;q22)	5 years F	no
Gadage V.S. et al. (2012) [48]	SM-AHN (AML)	D816V: ND	t(8;21)(q22;q22)	14 years F	no
Gogia A. et al. (2013) [53]	SM-AHN (AML)	Codon 816 (−)	46,XX	3 years F	maculopapular lesions
Rabade N. et al. (2016) [54]	SM-AHN (AML)	D816V (−)	t(8;21)(q22;q22)	7 years F	no
Mitchell S.G. et al. (2017) [57]	SM-AHN (CMML)	D816H (+)	49,XX,+8+8, der(8)t(1;8)(q21;p11.2), +12,i(12)(p10)	13 years F	no
Huang A. et al. (2017) [58]	ASM	D816V (+)	ND	1 month M	diffuse lesions
Zheng Y. et al. (2018) [55]	MCL	D816V (−)	47,XY+5,t(1;9)	13 years M	no

Abbreviations: BM—bone marrow; SM—systemic mastocytosis; AHN—hematologic neoplasm; F—female; M—male; AML—acute myeloid leukemia; CMML—chronic myelomonocytic leukemia; ASM- aggressive SM; ND—not done; (+)—positive result; (−)—negative result.

**Table 2:** SM reported in studies on selected groups of pediatric patients with mastocytosis.

Reference	Number of Patients	Number of SM Patients (%)	Form of SM
Bodemer C. et al. (2010) [19]	65	1 (1.5)	no data reported
Alvarez-Twose I. et al. (2012) [7]	111	2 (1.8)	1 ISM, 1 WDSM **
Lange M. et al. (2013) [12]	101	1 (1)	1 ISM
Méni C. et al. (2015) [8] *	1747	16 (0.9)	4 ISM; 8 MCS; 4 MCL
Carter M. et al. (2015) [9]	108	18 (16.6)	18 ISM
Matito A. et al. (2015) [125]	42	2 (4.8)	2 WDSM **
Méni C. et al. (2018) [16]	53	1 (1.9)	1 ISM
Carter M. et al. (2018) [10]	65	23 (35.4)	23 ISM
Czarny J. et al. (2020) [11] ***	32	4 (12.5)	1 SSM, 3 ISM

SM—systemic mastocytosis; ISM—indolent systemic mastocytosis; WDSM—well-differentiated SM; MCS—mast cell sarcoma; MCL—mast cell leukemia; * a systematic review of cases published between 1950 and 2014; ** the exact WHO type of SM was not provided; *** only children with cutaneous involvement >50% of body surface area were included.

**Table 3:** Examples of potential triggers of mast cell activation in patients with pediatric mastocytosis.

**Environmental and General Factors:**
Physical: friction, pressure, cold, heat, sudden temperature change
Nutrition: alcohol, caffeine, hot spices, rarely also fermented and matured foods (histamine-rich) *
Infectious diseases and fever (typically viral infections)
Teething
Emotional stress
Intensive exercise
**Allergens:**
Hymenoptera venoms and allergens that can be unique for the individual patient
(pollens, animal dander, molds, dust mite, food, among others)
**Drugs:**
Analgesics (i.e., aspirin, NSAIDs)
Opioids (i.e., morphine, codeine)
Muscle relaxants (i.e., atracurium, mivacurium, rocuronium)
Cough suppressants (i.e., dextromethorphan, codeine)
Contrast media (i.e., hyperosmolar and ionic contrast media)
Antibiotics (i.e., quinolones)
Vaccinations

* General dietary restrictions are not recommended and not necessary in most cases; NSAIDs—nonsteroidal anti-inflammatory drugs.

**Table 4:** Major treatment options for symptom control in pediatric mastocytosis.

Systems and Symptoms	First-Line Therapy	Other Therapeutic Options
Skin: pruritus flushing, blistering	HR1-antagonists	HR2-antagonists Oral corticosteroids (short course) Topical corticosteroids (class 1–3, short cycles ± occlusion) Leukotriene antagonist Pimecrolimus cream Topical sodium cromolyn Excision (for mastocytoma) NB-UVB * or PUVA ** Local care (for blistering)
Gastrointestinal: diarrhea, abdominal cramping/pain, reflux, ulceration	HR2-antagonists	Proton pump inhibitors Oral sodium cromolyn Oral corticosteroids
Neuro/psychiatric: headache, poor concentration, cognitive impairment	HR1 and HR2-antagonists	Neuro/psychiatric treatment specific for the individual patient according to symptoms
Cardiovascular: presyncope, syncope, hypotension	HR1 and HR2-antagonists	Oral corticosteroids Epinephrine
Osteopenia/osteoporosis	Calcium, vitamin D_3_	Treatment specific for the individual patient according to the age and T-score
Anaphylaxis	Epinephrine	Acute anaphylaxis: HR1 and HR2-antagonists Oral corticosteroids Intravenous fluids Prevention of anaphylaxis: Epinephrine auto-injector HR1-antagonists Allergen specific immunotherapy (typically Hymenoptera venom) Omalizumab (for recurrent episodes)

* NB-UVB—narrow-band ultraviolet B; ** PUVA—psoralen plus ultraviolet A.

## Data Availability

Not applicable.

## Abbreviations

AML	acute myeloid leukemia
ASM	aggressive systemic mastocytosis
BM	bone marrow
CM	cutaneous mastocytosis
CMML	chronic myelomonocytic leukemia
DCM	diffuse cutaneous mastocytosis
HR	histamine receptor
IgE	immunoglobulin E
ISM	indolent systemic mastocytosis
MCL	mast cell leukemia
MCS	mast cell sarcoma
MPCM	maculopapular cutaneous mastocytosis
NB-UVB	narrow-band ultraviolet B
NSAIDs	nonsteroidal anti-inflammatory drugs
PB	peripheral blood
PUVA	psoralen plus ultraviolet A
SM	systemic mastocytosis
SM-AHN	systemic mastocytosis with associated hematologic neoplasm
TKI	tyrosine kinase inhibitors
WDSM	well-differentiated SM
